# (*Z*)-*N*-[3-(4-Bromo­benzo­yl)-1,3-thia­zolidin-2-yl­idene]cyanamide

**DOI:** 10.1107/S1600536810046520

**Published:** 2010-11-17

**Authors:** Jiu-Ming Li, Jian-Ping Yong, Feng-lan Huang, Li-mei Sun, Ling Xu

**Affiliations:** a College of Chemistry and Chemical Engineering, Inner Mongolia University for Nationalities, Tongliao 028043, People’s Republic of China; b Inner Mongolia Industrial Engineering Research, Center of Universities for Castor, Tongliao 028042, People’s Republic of China; c School of Public Health, Ningxia Medical University, Yinchuan 750004, People’s Republic of China; d College of Life Science, Inner Mongolia University for Nationalities, Tongliao 028043, People’s Republic of China

## Abstract

In the title compound, C_11_H_8_BrN_3_OS, the dihedral angle between the benzene and thia­zolidine rings is 63.4 (2)°. Inter­molecular C—H⋯N inter­actions help to stabilize the crystal structure.

## Related literature

For related structures, see: Wang *et al.* (2008 ▶); Liu & Li (2009 ▶); Xie & Li (2010 ▶). For the biological activity of thia­zolidine-containing compounds, see: Iwata *et al.* (1988 ▶). For bond-length data, see: Allen *et al.* (1987 ▶).
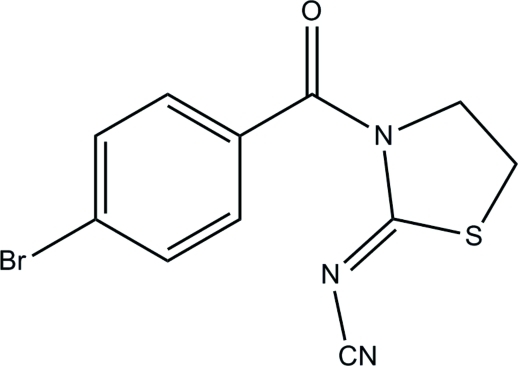


## Experimental

### 

#### Crystal data

C_11_H_8_BrN_3_OS
*M* *_r_* = 310.17
Monoclinic, 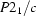

*a* = 16.579 (3) Å
*b* = 5.6471 (11) Å
*c* = 13.611 (3) Å
β = 112.91 (3)°
*V* = 1173.9 (4) Å^3^
*Z* = 4
Mo *K*α radiation
μ = 3.67 mm^−1^
*T* = 173 K
0.25 × 0.20 × 0.06 mm

#### Data collection

Rigaku Mercury CCD/AFC diffractometer
Absorption correction: multi-scan (*CrystalClear*; Rigaku, 2007 ▶) *T*_min_ = 0.461, *T*_max_ = 0.810
8232 measured reflections
2067 independent reflections
1960 reflections with *I* > 2σ(*I*)
*R*_int_ = 0.038

#### Refinement

*R*[*F* ^2^ > 2σ(*F* ^2^)] = 0.037
*wR*(*F* ^2^) = 0.108
*S* = 1.28
2067 reflections
154 parameters
H-atom parameters constrained
Δρ_max_ = 0.46 e Å^−3^
Δρ_min_ = −0.35 e Å^−3^

### 

Data collection: *CrystalClear* (Rigaku, 2007 ▶); cell refinement: *CrystalClear*; data reduction: *CrystalClear*; program(s) used to solve structure: *SHELXS97* (Sheldrick, 2008 ▶); program(s) used to refine structure: *SHELXL97* (Sheldrick, 2008 ▶); molecular graphics: *SHELXTL* (Sheldrick, 2008 ▶); software used to prepare material for publication: *SHELXTL*.

## Supplementary Material

Crystal structure: contains datablocks I, global. DOI: 10.1107/S1600536810046520/hg2746sup1.cif

Structure factors: contains datablocks I. DOI: 10.1107/S1600536810046520/hg2746Isup2.hkl

Additional supplementary materials:  crystallographic information; 3D view; checkCIF report

## Figures and Tables

**Table 1:** Hydrogen-bond geometry (Å, °)

*D*—H⋯*A*	*D*—H	H⋯*A*	*D*⋯*A*	*D*—H⋯*A*
C9—H9*A*⋯N3^i^	0.97	2.51	3.281 (5)	137

Symmetry code: (i) 
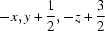
.

**Fig. 1.**
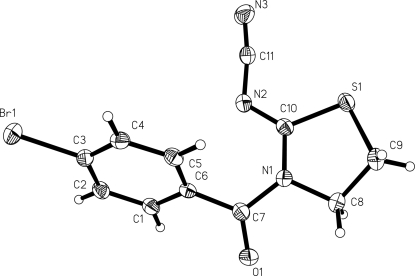
The molecular structure of (I), with atom labels and 40% probability displacement ellipsoids for non-H atoms.

## supplementary crystallographic information

### Comment

Thiazolidine is an important kind of group in organic chemistry. Many compounds containing thiazolidine groups possess a broad spectrum of biological activities (Iwata *et al.*, 1988). Here, we report the crystal structure of (I).

In title compound, all bond lengths in the molecular are normal (Allen *et al.*, 1987) and in a good agreement with those reported previously (Wang *et al.*, 2008; Liu & Li, 2009; Xie & Li, 2010). The dihedral angle between benzene (C1—C6) and thiazolidine (C8—C10/N1/S2) rings is 63.4 (2) °. The intermolecular C—H···N hydrogen bonds stabilize the structure.

### Experimental

A mixture of *N*-cyanoiminothiazolidine 10 mmol (1.27 g), 4-bromobenzoyl chloride (2.19 g, 10 mmol) and (1.01 g, 10 mmol) triethylamine was refluxed in absolute acetone (25 ml) for 3 h. On cooling, the product crystallized, was filtered, and recrystallized from absolute EtOH; yield 2.48 g (80.0%). Single crystals suitable for X-ray measurements were obtained by recrystallization from acetonitrile at room temperature.

### Refinement

H atoms were positioned geometrically and refined using a riding model, with C—H = 0.93 or 0.97 Å and with *U*_iso_(H) = 1.2 times *U*_eq_(C).

### Crystal data

**Table:**

C_11_H_8_BrN_3_OS	*F*(000) = 616
*M**_r_* = 310.17	*D*_x_ = 1.755 Mg m^−^^3^
Monoclinic, *P*2_1_/*c*	Mo *K*α radiation, λ = 0.71073 Å
Hall symbol: -P 2ybc	Cell parameters from 3665 reflections
*a* = 16.579 (3) Å	θ = 1.3–27.5°
*b* = 5.6471 (11) Å	µ = 3.67 mm^−^^1^
*c* = 13.611 (3) Å	*T* = 173 K
β = 112.91 (3)°	Plate, colorless
*V* = 1173.9 (4) Å^3^	0.25 × 0.20 × 0.06 mm
*Z* = 4	

### Data collection

**Table:**

Rigaku Mercury CCD/AFC diffractometer	2067 independent reflections
Radiation source: Sealed Tube	1960 reflections with *I* > 2σ(*I*)
Graphite Monochromator	*R*_int_ = 0.038
φ and ω scans	θ_max_ = 25.0°, θ_min_ = 1.3°
Absorption correction: multi-scan (*CrystalClear*; Rigaku, 2007)	*h* = −17→19
*T*_min_ = 0.461, *T*_max_ = 0.810	*k* = −6→6
8232 measured reflections	*l* = −16→13

### Refinement

**Table:**

Refinement on *F*^2^	Primary atom site location: structure-invariant direct methods
Least-squares matrix: full	Secondary atom site location: difference Fourier map
*R*[*F*^2^ > 2σ(*F*^2^)] = 0.037	Hydrogen site location: inferred from neighbouring sites
*wR*(*F*^2^) = 0.108	H-atom parameters constrained
*S* = 1.28	*w* = 1/[σ^2^(*F*_o_^2^) + (0.054*P*)^2^ + 0.437*P*] where *P* = (*F*_o_^2^ + 2*F*_c_^2^)/3
2067 reflections	(Δ/σ)_max_ = 0.001
154 parameters	Δρ_max_ = 0.46 e Å^−^^3^
0 restraints	Δρ_min_ = −0.35 e Å^−^^3^

### Special details

**Table:**

Geometry. All e.s.d.'s (except the e.s.d. in the dihedral angle between two l.s. planes) are estimated using the full covariance matrix. The cell e.s.d.'s are taken into account individually in the estimation of e.s.d.'s in distances, angles and torsion angles; correlations between e.s.d.'s in cell parameters are only used when they are defined by crystal symmetry. An approximate (isotropic) treatment of cell e.s.d.'s is used for estimating e.s.d.'s involving l.s. planes.
Refinement. Refinement of *F*^2^ against ALL reflections. The weighted *R*-factor *wR* and goodness of fit *S* are based on *F*^2^, conventional *R*-factors *R* are based on *F*, with *F* set to zero for negative *F*^2^. The threshold expression of *F*^2^ > σ(*F*^2^) is used only for calculating *R*-factors(gt) *etc*. and is not relevant to the choice of reflections for refinement. *R*-factors based on *F*^2^ are statistically about twice as large as those based on *F*, and *R*- factors based on ALL data will be even larger.

### Fractional atomic coordinates and isotropic or equivalent isotropic displacement parameters (Å^2^)

**Table:**

	*x*	*y*	*z*	*U*_iso_*/*U*_eq_	
Br1	0.46599 (2)	0.71386 (7)	0.56642 (3)	0.03845 (19)	
S1	0.07775 (6)	0.52236 (16)	0.83336 (8)	0.0307 (2)	
O1	0.26798 (18)	1.1823 (4)	0.8767 (2)	0.0362 (6)	
N1	0.19890 (18)	0.8299 (5)	0.8536 (2)	0.0268 (6)	
N2	0.15701 (19)	0.6055 (6)	0.6966 (2)	0.0316 (7)	
N3	0.0788 (2)	0.2687 (6)	0.5834 (3)	0.0483 (10)	
C1	0.3110 (2)	1.0740 (6)	0.6926 (3)	0.0281 (7)	
H1A	0.2843	1.2215	0.6845	0.034*	
C2	0.3582 (2)	1.0153 (6)	0.6309 (3)	0.0288 (8)	
H2B	0.3614	1.1193	0.5796	0.035*	
C3	0.3999 (2)	0.7995 (6)	0.6478 (3)	0.0267 (8)	
C4	0.3960 (2)	0.6399 (6)	0.7235 (3)	0.0280 (8)	
H4A	0.4261	0.4969	0.7347	0.034*	
C5	0.3469 (2)	0.6969 (6)	0.7817 (3)	0.0274 (8)	
H5A	0.3427	0.5903	0.8315	0.033*	
C6	0.3036 (2)	0.9141 (6)	0.7661 (3)	0.0251 (7)	
C7	0.2562 (2)	0.9906 (6)	0.8339 (3)	0.0276 (8)	
C8	0.1669 (2)	0.8942 (7)	0.9375 (3)	0.0323 (8)	
H8A	0.1231	1.0182	0.9126	0.039*	
H8B	0.2148	0.9492	1.0011	0.039*	
C9	0.1273 (2)	0.6690 (7)	0.9608 (3)	0.0332 (8)	
H9A	0.0837	0.7059	0.9897	0.040*	
H9B	0.1723	0.5702	1.0114	0.040*	
C10	0.1498 (2)	0.6544 (6)	0.7864 (3)	0.0259 (7)	
C11	0.1122 (2)	0.4235 (7)	0.6401 (3)	0.0339 (8)	

### Atomic displacement parameters (Å^2^)

**Table:**

	*U*^11^	*U*^22^	*U*^33^	*U*^12^	*U*^13^	*U*^23^
Br1	0.0425 (3)	0.0437 (3)	0.0358 (3)	−0.00069 (16)	0.0225 (2)	−0.00648 (16)
S1	0.0292 (5)	0.0296 (5)	0.0375 (5)	−0.0023 (4)	0.0175 (4)	0.0020 (4)
O1	0.0422 (15)	0.0270 (14)	0.0456 (17)	−0.0066 (11)	0.0239 (13)	−0.0086 (12)
N1	0.0302 (16)	0.0262 (15)	0.0266 (16)	−0.0027 (12)	0.0139 (13)	−0.0015 (12)
N2	0.0337 (16)	0.0340 (17)	0.0317 (16)	−0.0060 (13)	0.0178 (14)	−0.0028 (13)
N3	0.039 (2)	0.053 (2)	0.061 (3)	−0.0111 (17)	0.0277 (19)	−0.0232 (19)
C1	0.0256 (17)	0.0238 (17)	0.035 (2)	−0.0017 (14)	0.0117 (15)	0.0009 (15)
C2	0.0318 (18)	0.0275 (18)	0.0287 (19)	−0.0046 (14)	0.0135 (16)	0.0031 (14)
C3	0.0254 (18)	0.0306 (19)	0.0246 (19)	−0.0041 (14)	0.0103 (15)	−0.0045 (14)
C4	0.0283 (18)	0.0237 (17)	0.0308 (19)	−0.0007 (14)	0.0101 (15)	−0.0020 (14)
C5	0.0295 (19)	0.0231 (17)	0.0283 (19)	−0.0017 (14)	0.0097 (16)	0.0054 (14)
C6	0.0236 (16)	0.0248 (17)	0.0261 (18)	−0.0061 (14)	0.0088 (14)	−0.0043 (14)
C7	0.0258 (17)	0.0257 (18)	0.0324 (19)	−0.0002 (13)	0.0125 (15)	0.0017 (14)
C8	0.0360 (19)	0.036 (2)	0.030 (2)	−0.0016 (16)	0.0185 (16)	−0.0030 (16)
C9	0.0301 (19)	0.041 (2)	0.032 (2)	−0.0001 (16)	0.0158 (17)	0.0038 (17)
C10	0.0239 (17)	0.0228 (16)	0.0311 (19)	0.0013 (14)	0.0106 (15)	0.0031 (14)
C11	0.0310 (19)	0.035 (2)	0.043 (2)	−0.0043 (16)	0.0229 (17)	−0.0055 (18)

### Geometric parameters (Å, °)

**Table:**

Br1—C3	1.899 (4)	C2—C3	1.376 (5)
S1—C10	1.729 (3)	C2—H2B	0.9300
S1—C9	1.806 (4)	C3—C4	1.389 (5)
O1—C7	1.208 (4)	C4—C5	1.375 (5)
N1—C10	1.379 (4)	C4—H4A	0.9300
N1—C7	1.412 (4)	C5—C6	1.396 (5)
N1—C8	1.480 (4)	C5—H5A	0.9300
N2—C10	1.302 (5)	C6—C7	1.491 (5)
N2—C11	1.325 (5)	C8—C9	1.520 (5)
N3—C11	1.154 (5)	C8—H8A	0.9700
C1—C6	1.388 (5)	C8—H8B	0.9700
C1—C2	1.392 (5)	C9—H9A	0.9700
C1—H1A	0.9300	C9—H9B	0.9700
			
C10—S1—C9	92.05 (17)	C1—C6—C7	118.3 (3)
C10—N1—C7	127.0 (3)	C5—C6—C7	121.7 (3)
C10—N1—C8	113.1 (3)	O1—C7—N1	118.7 (3)
C7—N1—C8	117.3 (3)	O1—C7—C6	122.1 (3)
C10—N2—C11	118.3 (3)	N1—C7—C6	119.1 (3)
C6—C1—C2	120.5 (3)	N1—C8—C9	105.6 (3)
C6—C1—H1A	119.7	N1—C8—H8A	110.6
C2—C1—H1A	119.7	C9—C8—H8A	110.6
C3—C2—C1	118.3 (3)	N1—C8—H8B	110.6
C3—C2—H2B	120.8	C9—C8—H8B	110.6
C1—C2—H2B	120.8	H8A—C8—H8B	108.7
C2—C3—C4	122.2 (3)	C8—C9—S1	104.8 (3)
C2—C3—Br1	119.7 (3)	C8—C9—H9A	110.8
C4—C3—Br1	118.1 (3)	S1—C9—H9A	110.8
C5—C4—C3	118.9 (3)	C8—C9—H9B	110.8
C5—C4—H4A	120.5	S1—C9—H9B	110.8
C3—C4—H4A	120.5	H9A—C9—H9B	108.9
C4—C5—C6	120.2 (3)	N2—C10—N1	122.0 (3)
C4—C5—H5A	119.9	N2—C10—S1	125.7 (3)
C6—C5—H5A	119.9	N1—C10—S1	112.2 (2)
C1—C6—C5	119.8 (3)	N3—C11—N2	171.8 (4)
			
C6—C1—C2—C3	−2.6 (5)	C1—C6—C7—N1	138.8 (3)
C1—C2—C3—C4	0.3 (5)	C5—C6—C7—N1	−47.2 (4)
C1—C2—C3—Br1	−179.3 (2)	C10—N1—C8—C9	30.9 (4)
C2—C3—C4—C5	1.7 (5)	C7—N1—C8—C9	−165.9 (3)
Br1—C3—C4—C5	−178.7 (2)	N1—C8—C9—S1	−35.6 (3)
C3—C4—C5—C6	−1.4 (5)	C10—S1—C9—C8	26.6 (3)
C2—C1—C6—C5	2.9 (5)	C11—N2—C10—N1	175.3 (3)
C2—C1—C6—C7	177.0 (3)	C11—N2—C10—S1	−7.3 (5)
C4—C5—C6—C1	−0.8 (5)	C7—N1—C10—N2	5.6 (5)
C4—C5—C6—C7	−174.7 (3)	C8—N1—C10—N2	166.7 (3)
C10—N1—C7—O1	151.9 (3)	C7—N1—C10—S1	−172.1 (3)
C8—N1—C7—O1	−8.6 (5)	C8—N1—C10—S1	−11.0 (4)
C10—N1—C7—C6	−31.3 (5)	C9—S1—C10—N2	172.4 (3)
C8—N1—C7—C6	168.2 (3)	C9—S1—C10—N1	−10.0 (3)
C1—C6—C7—O1	−44.6 (5)	C10—N2—C11—N3	−171 (3)
C5—C6—C7—O1	129.4 (4)		

### Hydrogen-bond geometry (Å, °)

**Table:**

*D*—H···*A*	*D*—H	H···*A*	*D*···*A*	*D*—H···*A*
C9—H9A···N3^i^	0.97	2.51	3.281 (5)	137

Symmetry codes: (i) −*x*, *y*+1/2, −*z*+3/2.
